# Low diffusion capacity predicts poor prognosis in extensive stage small cell lung cancer: a single-center analysis of 10 years

**DOI:** 10.1007/s00432-023-04686-2

**Published:** 2023-03-13

**Authors:** Jee Seon Kim, Eun Ji Kim, Jong Geol Jang, Kyung Soo Hong, June Hong Ahn

**Affiliations:** 1 Division of Pulmonology, Department of Internal Medicine, Pohang Semyeong Christianity Hospital, Pohang, Republic of Korea; 2 Division of Pulmonology and Allergy, Department of Internal Medicine, College of Medicine, Yeungnam University and Respiratory Center, Yeungnam University Medical Center, 170 Hyeonchung-Ro, Namgu, Daegu, 42415 Republic of Korea

**Keywords:** Diffusion capacity (DLco), Prognosis, Small-cell lung cancer (SCLC)

## Abstract

**Background:**

Poor pulmonary function and chronic obstructive pulmonary disease (COPD) are associated with poorer overall survival (OS) in non-small-cell lung cancer (NSCLC) patients. Few studies have investigated the association between pulmonary function and OS in small-cell lung cancer (SCLC) patients. We compared the clinical characteristics of extensive disease SCLC (ED-SCLC) with or without moderately impaired diffusion capacity for carbon monoxide (DLco) and investigated the factors associated with survival in ED-SCLC patients.

**Methods:**

This retrospective single-center study was performed between January 2011 and December 2020. Of the 307 SCLC patients who received cancer therapy during the study, 142 with ED-SCLC were analyzed. The patients were divided into DLco < 60% group and DLco ≥ 60% groups. OS and predictors of poor OS were analyzed.

**Results:**

The median OS of the 142 ED-SCLC patients was 9.3 months and the median age was 68 years. In total, 129 (90.8%) patients had a history of smoking, and 60 (42.3%) had COPD. Thirty-five (24.6%) patients were assigned to the DLco < 60% group. Multivariate analysis revealed that DLco < 60% (odds ratio [OR], 1.609; 95% confidence interval [CI], 1.062–2.437; P = 0.025), number of metastases (OR, 1.488; 95% CI, 1.262–1.756; P < 0.001), and < 4 cycles of first-line chemotherapy (OR, 3.793; 95% CI, 2.530–5.686; P < 0.001) were associated with poor OS. Forty (28.2%) patients received < 4 cycles of first-line chemotherapy; the most common reason for this was death (n = 22, 55%) from grade 4 febrile neutropenia (n = 15), infection (n = 5), or massive hemoptysis (n = 2). The DLco < 60% group had a shorter median OS than the DLco ≥ 60% group (10.6 ± 0.8 vs. 4.9 ± 0.9 months, P = 0.003).

**Conclusions:**

In this study, approximately one quarter of the ED-SCLC patients had DLco < 60%. Low DLco (but not forced expiratory volume in 1 s or forced vital capacity), a large number of metastases, and < 4 cycles of first-line chemotherapy were independent risk factors for poor survival outcomes in patients with ED-SCLC.

## Introduction

Lung cancer is the leading cause of cancer deaths worldwide; there were an estimated 1.8 million deaths in 2020 (Jeon et al. 2015). Small-cell lung cancer (SCLC) is a smoking-related disease. SCLC represents approximately 15% of all lung cancers and is characterized by a rapid doubling time and early development of widespread metastases (Rudin et al. 2021). The overall mortality rate of SCLC has decreased in high-income countries as a result of a declining incidence of smoking (Rudin et al. 2021; Howlader et al. 2020). However, due to limited improvements in the treatment of SCLC, cancer-specific survival has remained low over the past two decades; the 2-year survival is 10% among men and 15% among women in the USA (Howlader et al. 2020; Paesmans et al. 2000). Extensive disease SCLC (ED-SCLC) comprises about two-thirds of SCLC cases and most patients die within 1 year despite initial chemotherapy response rates > 60% (Rudin et al. 2021).

Poor prognostic factors for survival of SCLC include older age, male sex, poor performance status, extensive disease, weight loss, and elevated lactate dehydrogenase (LDH) activity (Paesmans et al. 2000; Hong et al. 2010; Ma et al. 2021; Liu et al. 2015; Ganti et al. 2021; Albain et al. 1990). Younger age, good performance status, normal LDH activity, normal creatinine level, and a single metastatic site are favorable prognostic factors in patients with ED-SCLC (Ganti et al. 2021; Albain et al. 1990; Foster et al. 2009).

Preoperative spirometry predicts postoperative morbidity and mortality, and the diffusion capacity for carbon monoxide (DLco) is associated with long-term survival in non-small-cell lung cancer (NSCLC) patients (Ferguson et al. 2012; Brunelli et al. 2013). An association between pulmonary function and overall survival (OS) of SCLC patients has been reported (Videtic et al. 2004; Kang et al. 2018). According to Videtic et al. (Videtic et al. 2004), DLco < 60% is associated with toxicity-related treatment interruptions and decreased survival in limited-disease SCLC. Kang et al. (2018) reported that forced expiratory volume in 1 s (FEV_1_) < 80% was an independent prognostic factor in patients with ED-SCLC. However, no study has analyzed the association between DLco and survival in ED-SCLC patients. Thus, we compared the clinical characteristics of ED-SCLC patients with and without an impaired DLco and investigated the factors associated with survival.

## Methods

### Study design and subjects

This retrospective observational study was performed between January 2011 and December 2020 at Yeungnam University Hospital, which is a 930-bed, university-affiliated tertiary referral hospital in Daegu, South Korea. Of the 307 SCLC patients who received cancer therapy during the study, 142 with ED-SCLC were analyzed. All patients with pathologically confirmed ED-SCLC and no history of treatment were included. Patients who lacked pulmonary function test (PFT) data and were untreated were excluded.

### Diagnostic procedure and pulmonary function testing

The staging procedure included routine laboratory tests, chest radiography, chest computed tomography (CT), a whole-body bone scan, 18F-fluorodeoxyglucose positron emission tomography-CT, and brain magnetic resonance imaging (MRI).

The PFTs were performed at the time of the lung cancer diagnosis following the 2005 American Thoracic Society and European Respiratory Society criteria (Miller et al. 2005; MacIntyre et al. 2005). Forced vital capacity (FVC) and FEV_1_ were determined from a flow-volume curve drawn using a spirometer. The largest value from a minimum of three maneuvers was recorded (Miller et al. 2005). DLco was measured by single-breath diffusing capacity maneuvers during at least two valid tests (MacIntyre et al. 2005). PFT values are reported as percent predicted. Normal values of FVC, FEV_1_ and DLco were calculated using the method described by Choi et al. (2005) and the European Community for Steel and Coal (Quanjer 1983).

### Data collection

We extracted the following data from the medical records: age, sex, body mass index (BMI), Eastern Cooperative Oncology Group performance status (ECOG PS), smoking history, PFT results, underlying diseases, metastatic sites, diagnosis to treatment interval, history of chemotherapy, first-line chemotherapy regimen, number of cycles, treatment response, survival status, and the date of death or last follow-up visit. Underlying comorbidities such as chronic obstructive pulmonary disease (COPD), diabetes mellitus, and hypertension were also investigated. The response to chemotherapy was evaluated by a CT scan (and brain MRI if a brain metastasis was present) after every two cycles, following the Response Evaluation Criteria in Solid Tumors (RECIST) ver.1.1 criteria (Eisenhauer et al. 2009).

### Statistical analyses

Patients were divided into DLco < 60% and DLco ≥ 60% groups. OS and predictors of a poor OS were analyzed. OS was defined as the time between the pathological diagnosis and date of death or last follow-up visit.

Student’s *t*-test and the Mann–Whitney *U* test were used to compare continuous variables. Pearson’s chi-square test was used to compare categorical variables and the results are expressed as frequencies (percentages). Univariate and multivariate analyses, the latter of which included factors with a P-value < 0.1 in univariate analysis, and Cox proportional hazards regression analyses were performed to identify prognostic factors for OS. Odds ratios (ORs) and their corresponding 95% confidence intervals (CIs) were calculated for predictors that were significant in the multivariate analysis. Survival probability was calculated using Kaplan–Meier analyses and compared using the log-rank test. A two-sided P-value < 0.05 was considered significant for all tests. All statistical procedures were performed with SPSS software (version 24.0; IBM Corp., Armonk, NY, USA).

### Ethics statement

This study was conducted following the tenets of the Declaration of Helsinki, and the protocol was reviewed and approved by the Institutional Review Board of Yeungnam University Hospital (YUH IRB 2022–08–016). The requirement for informed consent was waived because of the retrospective study design.

## Results

### Clinical characteristics

The baseline characteristics of the 142 ED-SCLC patients are presented in Table 1. The median age was 68 years (range: 50–85 years) and the majority were men (88.0%). The median BMI was 22.8 kg/m^2^ and 111 patients (78.2%) had an ECOG PS of 0–1. In total, 129 (90.8%) patients had a history of smoking, and 60 (42.3%) had COPD. Fourteen (9.9%) patients had no metastases, and most patients (90.1%) had at least one metastatic organ. The most common metastatic organ was bone, followed by the pleura, liver, brain, and adrenal glands. The mean period from diagnosis to treatment was 11 days (range: 0–91 days). Most patients received the platinum-based doublet as first-line chemotherapy (93.7%) and the most common regimen was etoposide plus cisplatin (EP) (77.5%), followed by etoposide plus carboplatin (EC) (14.1%) and irinotecan plus cisplatin (IP) (2.1%). The median number of cycles of first-line chemotherapy was 5 (range: 1–13). The overall response and disease control rates for first-line chemotherapy were 27.4% and 87.3%, respectively.

**Table 1:** Baseline characteristics of the ED-SCLC patients

Variables	Total (n = 142)	DLco ≥ 60% group (n = 107)	DLco < 60% group (n = 35)	P value
Age, years	68 (50–85)	68 (50–85)	70 (52–85)	0.120
Male	125 (88.0)	94 (87.9)	31 (88.6)	1.000
BMI (kg/m^2^)	22.8 (17.2–31.1)	22.8 (17.2–31.1)	22.9 (17.4–28.4)	0.504
ECOG PS				0.390
0	37 (26.1)	25 (23.4)	12 (34.3)	
1	74 (52.1)	58 (54.2)	16 (45.7)	
2	28 (19.7)	22 (20.6)	6 (17.1)	
3	3 (2.1)	2 (1.9)	1 (2.9)	
Smoking				0.304
Never smoker	13 (9.2)	11 (10.3)	2 (5.7)	
Ex-smoker	53 (37.3)	41 (38.3)	12 (34.3)	
Current smoker	76 (53.5)	55 (51.4)	21 (60.0)	
Pulmonary function test				
Percent predicted FVC	78 (45–130)	82 (45–130)	66 (46–109)	< 0.001
Percent predicted FEV_1_	75 (44–150)	78 (44–150)	73 (60–144)	< 0.001
Percent predicted DLco	67 (33–144)	73 (60–144)	52 (33–59)	< 0.001
Comorbidities				
COPD	60 (42.3)	47 (43.9)	13 (37.1)	0.481
DM	38 (26.8)	30 (28.0)	8 (22.9)	0.548
HTN	69 (48.6)	53 (49.5)	16 (45.7)	0.695
Number of metastatic organs				
0	14 (9.9)	11 (10.3)	3 (8.6)	0.050
1	56 (39.4)	46 (43.0)	10 (28.6)	
2	35 (24.6)	28 (26.2)	7 (20.0)	
3	24 (16.9)	13 (12.1)	11 (31.4)	
4	13 (9.2)	9 (8.4)	4 (11.4)	
Metastatic organs				
Liver	48 (33.8)	33 (30.8)	15 (42.9)	0.192
Brain	34 (23.9)	27 (25.2)	7 (20.0)	0.529
Adrenal gland	23 (16.2)	16 (15.0)	7 (20.0)	0.482
Bone	75 (52.8)	53 (49.5)	22 (62.9)	0.170
Pleura	50 (35.2)	35 (32.7)	15 (42.9)	0.275
Diagnosis to treatment interval, days	11 (0–91)	11 (0–91)	10 (1–83)	0.785
First-line chemotherapy regimen				0.448
EP	110 (77.5)	81 (75.7)	29 (82.9)	
EC	20 (14.1)	17 (15.9)	3 (8.6)	
IP	3 (2.1)	1 (0.9)	2 (5.7)	
Others	9 (6.3)	8 (7.4)	1 (2.9)	
First-line chemotherapy cycles	5 (1–13)	5 (1–13)	4 (1–9)	0.005
< 4 cycles of first-line chemotherapy	40 (28.2)	23 (21.9)	17 (48.6)	0.002
Response to first-line chemotherapy				0.084
CR	8 (5.6)	3 (2.8)	5 (14.3)	
PR	31 (21.8)	25 (23.4)	6 (17.1)	
SD	85 (59.9)	67 (62.6)	18 (51.4)	
PD	5 (3.5)	3 (2.8)	2 (5.7)	
Not evaluable	13 (9.2)	9 (8.4)	4 (11.4)	

Data are presented as median (range) or number (%)

BMI, body mass index; COPD, chronic obstructive pulmonary disease; CR, complete response; DLco, diffusion capacity for carbon monoxide; DM, diabetes mellitus; EC, etoposide + carboplatin; ECOG PS, Eastern Cooperative Oncology Group performance status; ED-SCLC, extensive disease small-cell lung cancer; EP, etoposide + cisplatin; FEV_1_, forced expiratory volume in 1 s; FVC, forced vital capacity; HTN, hypertension; IP, irinotecan + cisplatin; PD, progressive disease; PR, partial response; SD, stable disease

Among the patients, 35 (24.6%) were in the DLco < 60% group (median, 52%; range: 33–59%) (Table 1). The DLco < 60% group had a lower FVC (82% vs. 66%, P < 0.001), lower FEV_1_ (78% vs. 73%, P < 0.001), and underwent fewer first-line chemotherapy cycles (5 vs. 4, P = 0.005) than the DLco ≥ 60% group. The proportion of patients who received < 4 cycles of first-line chemotherapy was significantly higher in the in the DLco < 60% than ≥ 60% group (48.6% vs. 21.9%, P = 0.002). The number of metastatic organs tended to be higher in the DLco < 60% group (P = 0.050).

### Prognostic factors associated with overall survival

**Fig. 1:**
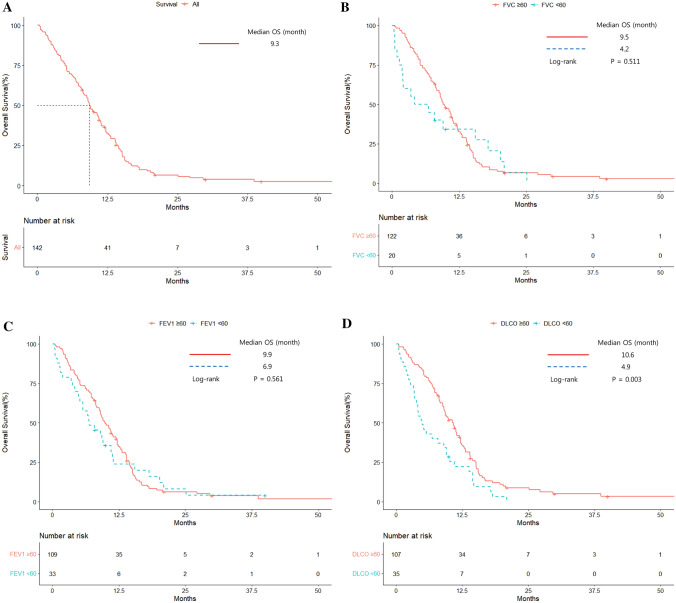
Kaplan–Meier overall survival curves. **A** Overall survival of all patients. **B** Overall survival according to forced vital capacity. **C** Overall survival according to forced expiratory volume in 1 s. **D** Overall survival according to diffusion capacity for carbon monoxide

The median OS of the 142 ED-SCLC patients was 9.3 months. The OS rates estimated by the Kaplan–Meier method were 28.8%, 4.9%, and 0.7% at 12.5, 25, and 50 months, respectively (Fig. 1A). The median OS rates did not differ by FVC (≥ 60% vs. < 60% group) or FEV_1_ (≥ 60% vs. < 60% group) (9.5 vs. 4.2 months, P = 0.511; and 9.9 vs. 6.9 months, P = 0.561, respectively) (Fig. 1B and C). However, the DLco < 60% group had a shorter median OS than the DLco ≥ 60% group (10.6 ± 0.8 vs. 4.9 ± 0.9 months, P = 0.003) (Fig. 1D).

Univariate analysis showed that DLco < 60% (OR, 1.809; 95% CI, 1.218–2.687, P = 0.003), the number of metastatic organs (OR, 1.453; 95% CI, 1.232–1.714, P < 0.001), liver metastasis (OR, 2.049; 95% CI, 1.416–2.966, P < 0.001), bone metastasis (OR, 1.841; 95% CI, 1.290–2.626, P = 0.001), first-line chemotherapy EP regimen (OR, 0.646; 95% CI, 0.414–1.008, P = 0.054) and < 4 cycles of first-line chemotherapy (OR, 3.805; 95% CI, 2.587–5.597, P < 0.001) were poor prognostic factors for OS (Table 2).

**Table 2:** Cox regression analyses of overall survival in patients with ED-SCLC

Variables	Univariate			Multivariate		
	OR	95% CI	P-value	OR	95% CI	P-value
Male	1.543	0.905–2.631	0.111			
Aged ≥ 65 years	1.136	0.793–1.629	0.487			
BMI	1.104	0.957–1.073	0.643			
ECOG PS ≥ 2	1.118	0.744–1.679	0.592			
Current or ex-smoker	1.447	0.785–2.665	0.236			
Pulmonary function test						
FVC < 60%	1.183	0.714–1.958	0.514			
FEV_1_ < 60%	1.130	0.745–1.714	0.564			
DLco < 60%	1.809	1.218–2.687	0.003	1.609	1.062–2.437	0.025
Comorbidities						
COPD	1.024	0.721–1.457	0.893			
DM	1.307	0.894–1.913	0.167			
HTN	1.174	0.830–1.660	0.366			
Number of metastatic organs	1.453	1.232–1.714	< 0.001	1.488	1.262–1.756	< 0.001
Liver metastasis	2.049	1.416–2.966	< 0.001			
Brain metastasis	1.271	0.855–1.889	0.236			
Adrenal metastasis	1.502	0.945–2.388	0.085			
Bone metastasis	1.841	1.290–2.626	0.001			
Pleural metastasis	1.018	0.705–1.472	0.923			
Diagnosis to treatment interval, days	1.004	0.990–1.018	0.617			
First-line chemotherapy EP regimen	0.646	0.414–1.008	0.054			
First-line chemotherapy cycles < 4	3.805	2.587–5.597	< 0.001	3.793	2.530–5.686	< 0.001

BMI, body mass index; CI, confidence interval; COPD, chronic obstructive pulmonary disease; DLco, diffusion capacity for carbon monoxide; DM, diabetes mellitus; ECOG PS, Eastern Cooperative Oncology Group performance status; ED-SCLC, extensive disease small cell lung cancer; EP, etoposide + cisplatin; FEV_1_, forced expiratory volume in 1 s; FVC, forced vital capacity; HTN, hypertension; OR, odds ratio

**Fig. 2:**
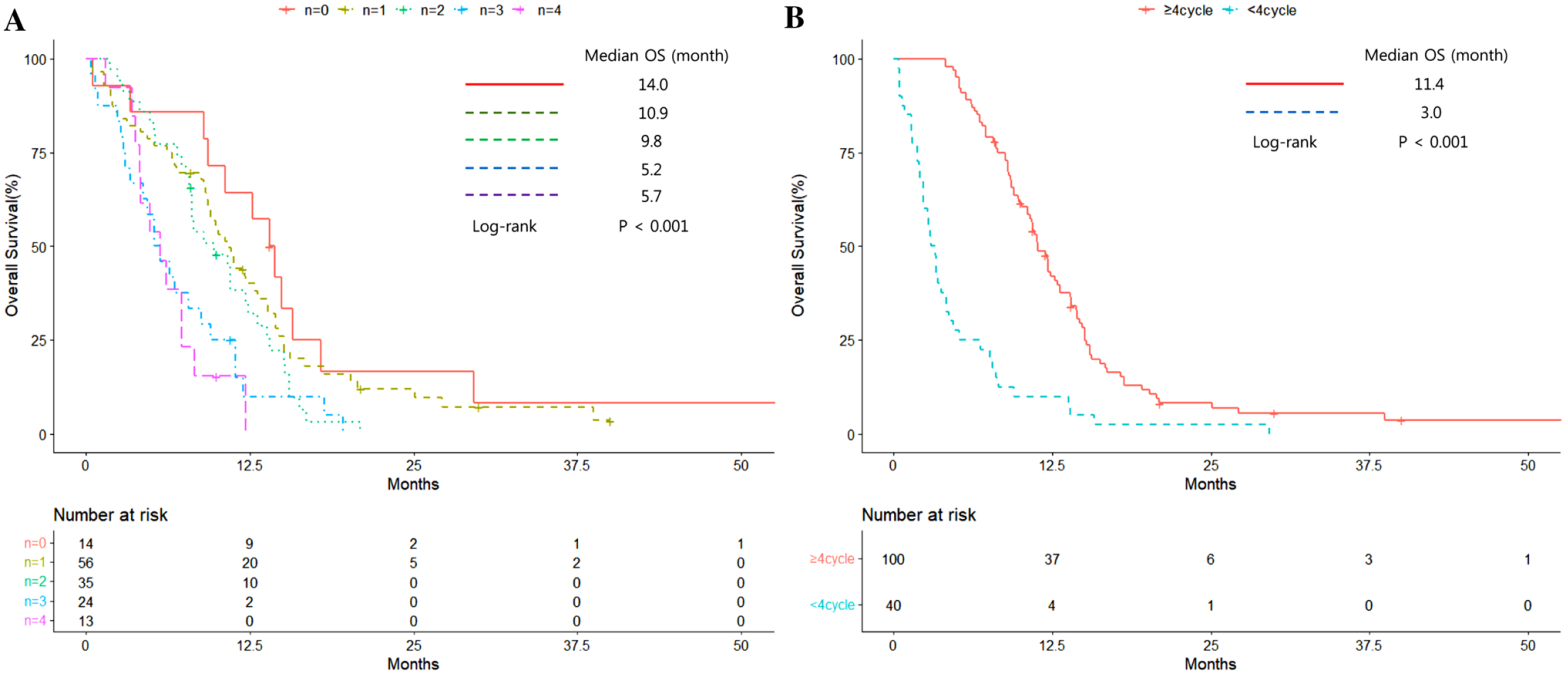
Kaplan–Meier overall survival curves according to **A** the number of metastatic organs and **B** number of first-line chemotherapy cycles. **A** Overall survival according to the number of metastatic organs. **B** Overall survival according to the number of first-line chemotherapy cycles

The multivariate analysis revealed that DLco < 60% (OR, 1.609; 95% CI, 1.062–2.437; P = 0.025), more metastatic sites (OR, 1.488; 95% CI, 1.262–1.756; P < 0.001), and < 4 cycles of first-line chemotherapy (OR, 3.793; 95% CI, 2.530–5.686; P < 0.001) were associated with a worse OS (Table 2). The Kaplan–Meier curves indicated that more metastatic organs was associated with a poor prognosis; as the number of metastatic organs increased, the survival rate decreased (no metastasis vs. four metastatic organs: median OS, 14.0 vs. 5.7 months, P < 0.001) (Fig. 2A). The < 4 cycles of chemotherapy group had a shorter median OS than the chemotherapy ≥ 4 cycles group (11.4 vs. 3.0 months, P < 0.001) (Fig. 2B).

### Reasons for incomplete first-line chemotherapy

In total, 40 of 142 (28.2%) patients received < 4 cycles of first-line chemotherapy; the most common reason for this was death (n = 22/40, 55%) from grade 4 febrile neutropenia (n = 15), infection (n = 5), or massive hemoptysis (n = 2) (Table 3). Other reasons for not completing first-line chemotherapy were a decrease in ECOG PS (n = 8), disease progression (n = 5), and adverse events during chemotherapy (n = 5) such as neutropenia and hepatitis.

**Table 3:** Reasons for < 4 cycles of first-line chemotherapy

Cause	Total (n = 40)
Death	22 (55)
Febrile neutropenia, grade 4	15 (37.5)
Infection	5 (12.5)
Massive hemoptysis	2 (5)
Decreased ECOG PS	8 (20)
Progressive disease	5 (12.5)
Adverse event of chemotherapy	5 (12.5)
Neutropenia	3 (7.5)
Hepatitis	2 (5)

Data are presented as numbers (%)

ECOG PS, Eastern Cooperative Oncology Group performance status

## Discussion

In this study, we identified several clinical factors associated with the prognosis of ED-SCLC, including low DLco, large number of metastatic organs, and < 4 cycles of first-line chemotherapy. Our analysis showed that a lower DLco was an independent predictor of survival in patients with ED-SCLC. However, neither FEV_1_ nor FVC was associated with a poor prognosis of ED-SCLC. To our knowledge, this is the first study to identify a relationship between DLco and the prognosis of ED-SCLC.

DLco is a measure of gas transfer that reflects the complex interactions that occur at the alveolar-capillary interface. A low DLco is associated with destruction of the airspace secondary to emphysema and a lower pulmonary vascular volume (Balasubramanian et al. 2019). DLco provided insight into functional limitations in patients with COPD and lung cancer (Videtic et al. 2004; Balasubramanian et al. 2019; de-Torres et al. 2021). A low DLco is associated with reduced exercise performance, severe exacerbations, and all-cause mortality in patients with COPD (Balasubramanian et al. 2019; de-Torres et al. 2021).

DLco reflects the functional status of lung cancer patients and is a general indicator of patient performance (Ferguson et al. 1988, 2012; Brunelli et al. 2013; Videtic et al. 2004). A low preoperative DLco is a predictor of postoperative cardiopulmonary complications, mortality, and poor long-term survival in surgical patients, including those with a normal FEV_1_ (Ferguson et al. 1988, 2012; Brunelli et al. 2013). According to Videtic et al. (2004), a low DLco is a marker of treatment tolerance and poor OS in patients with limited-disease SCLC. However, Lee et al. (2019) reported that a low DLco was not associated with poor survival in patients with ED-SCLC. In our study, the DLco < 60% group underwent fewer first-line chemotherapy cycles compared with the DLco ≥ 60% group (5 vs. 4, P = 0.005). The proportion of patients who received < 4 cycles of first-line chemotherapy was significantly higher in the DLco < 60% than ≥ 60% group (48.6% vs. 21.9%, P = 0.002). The low DLco may have affected the treatment tolerance of ED-SCLC patients, and the relatively few chemotherapy cycles may have affected their survival.

Lee et al. (2021) analyzed the Korean Health Insurance Review and Assessment Service database and reported that COPD increases the risk of death 1.17-fold in ED-SCLC patients. Another study noted that FEV_1_ < 80% was associated with shorter survival in patients with ED-SCLC (Kang et al. 2018). However, COPD and airflow limitation were not associated with survival in our study.

The number of metastatic sites at baseline is the most important prognostic predictor for OS in patients with ED-SCLC (Albain et al. 1990; Foster et al. 2009), and patients who have ≥ 2 metastatic sites have a significantly worse OS (Foster et al. 2009). Hong et al. (2010) confirmed that the disease extent, including liver metastasis, is a poor prognostic factor for SCLC. In an analysis of real-world data from 988 SCLC patients, Ma et al. (2021) showed that ED-SCLC without liver, bone, or subcutaneous metastases has favorable clinical outcomes. Our study confirmed that a high disease burden, i.e., more metastatic organs, was an independent risk factor for short OS in patients with ED-SCLC.

The standard treatment for ED-SCLC over the past two decades has been 4–6 cycles of a platinum-based etoposide regimen (Ganti et al. 2021). Liu et al. (2015) reported that ≥ 4 chemotherapy cycles (OR, 0.486; 95% CI, 0.301–0.786, P = 0.003) was a favorable prognostic factor for OS in SCLC patients. Other studies also reported that < 4 cycles of first-line chemotherapy predicted a shorter survival time in patients with ED-SCLC (Lee et al. 2019; Kim et al. 2022). However, some SCLC patients cannot undergo four cycles of full-dose chemotherapy because of old age or poor performance status (Kim et al. 2022). Kim et al. (2022) reported that first-line EP dose-reduced chemotherapy offered no significant survival disadvantage over full-dose chemotherapy in elderly ED-SCLC patients if they received a minimum of four cycles (median OS, 10.9 vs. 9.4 months, P = 0.817). Thus, a minimum of four cycles of dose-reduced chemotherapy should be considered in patients with SCLC who cannot tolerate full-dose chemotherapy.

First-line chemotherapy EP showed a trend toward good OS in the univariate analysis of our study, but was not associated with good OS in the multivariate analysis. In East Asian studies, IP as first-line chemotherapy improved survival compared with EP (Noda and Saijo 2002), but no significant difference was reported in western populations (Lara et al. 2009). According to a Korean nationwide cohort study (Lee et al. 2021), patients who receive IP have better survival outcomes than those who receive etoposide-based platinum therapy. In our study, no significant prognostic differences were detected between chemotherapy drug regimens.

The overall response rate to first-line chemotherapy in our study (27.4%) was lower than previous studies (Hong et al. 2010; Ma et al. 2021). Although the RECIST ver. 1.1 represents an evolution of these radiographic criteria, it relies on human measurement (Villaruz and Socinski 2013). The median OS (9.3 months), and disease control rate to first-line chemotherapy (87.3%) in our study were similar or superior compared to previous studies (Hong et al. 2010; Ma et al. 2021). Therefore, the difference in overall response rate is seen as a problem simply due to the difference in measurement between researchers.

This study had several limitations. First, it was a retrospective, observational single-center study with a small number of patients, although 10 years of medical records were available. However, considering the low prevalence of SCLC, it is not a number to be underestimated. Second, we did not evaluate potential confounding factors, such as hemoglobin, emphysema, destructive tuberculosis changes, pneumoconiosis, and pulmonary fibrosis. Given the high prevalence and morbidity of tuberculosis in Korea, the impact of these factors on DLco may be considerable. Finally, the prognosis of patients treated with immune checkpoint inhibitors was not analyzed. Adding an immune checkpoint inhibitor to platinum-based doublet therapy is the new standard of care for first-line treatment of ED-SCLC (Ganti et al. 2021). In South Korea, atezolizumab combined with EC therapy has only been available since August 1, 2020, so the effect of immunotherapy could not be assessed. Nonetheless, to our knowledge, this is the first study showing that a low DLco is associated with a poor prognosis in patients with ED-SCLC. Further prospective cohort studies are needed to verify whether DLco is a poor prognostic factor in SCLC patients.

## Conclusions

In this study, approximately one quarter of ED-SCLC patients had DLco < 60%. Lower DLco (but not FEV_1_ or FVC), a large number of metastases, and < 4 cycles of first-line chemotherapy were independent risk factors for poor survival outcomes in ED-SCLC patients. Strategies to ensure completion of ≥ 4 cycles of first-line chemotherapy are needed to improve OS in patients with ED-SCLC.

## Data Availability

The data set supporting the conclusions of this article is available from the corresponding author upon reasonable request.
